# Feeding Habits and the Occurrence of Anthropogenic Debris in the Stomach Content of Marine Fish from Pattani Bay, Gulf of Thailand

**DOI:** 10.3390/biology11020331

**Published:** 2022-02-19

**Authors:** Kay Khine Soe, Sukree Hajisamae, Penjai Sompongchaiyakul, Prawit Towatana, Siriporn Pradit

**Affiliations:** 1 Faculty of Environmental, Prince of Songkla University, Songkhla 90110, Thailand; 6010033001@email.psu.ac.th (K.K.S.); prawit.t@psu.ac.th (P.T.); 2 Department of Marine Science, Myeik University, Myeik 14051, Myanmar; 3 Faculty of Science and Technology, Prince of Songkla University, Pattani 94000, Thailand; sukree.h@psu.ac.th; 4 Department of Marine Science, Chaulalongkorn University, Bangkok 10330, Thailand; penjai.s@chula.ac.th; 5 Coastal Oceanography and Climate Change Research Center, Prince of Songkla University, Songkhla 90110, Thailand

**Keywords:** feeding features, microplastic, food type, season, water depth

## Abstract

**Simple Summary:**

In this work, the feeding behaviour of fish from a natural bay environment and the ingested anthropogenic fragments in a fish community in relation to their feeding habits and habitats were investigated. The identification of 34 fish species and analysis of their stomach content by visual inspection were carried out. The ingestion of anthropogenic debris by fish differed between season and their feeding features. The planktivorous fish having higher ingestion of anthropogenic debris than other species were found. The study results enhance the understanding of the spatiotemporal variation of feeding habits of fish communities and support future alerts relating to the risk of anthropogenic pollution in marine food webs.

**Abstract:**

This study assessed the feeding habits and ingestion of anthropogenic debris in 34 marine fish species from the southern Gulf of Thailand. A total of 5478 fish samples of 12 families were categorised into seven groups: planktivore, *Lucifer* feeder, fish feeder, *Acetes* feeder, shrimp feeder, piscivore, and zoobenthivore fish. A total of 2477 anthropogenic debris items were extracted from 12 fish species by visual inspection. Their ingestion of anthropogenic debris was influenced by season (*p* < 0.0001), with the highest ingestion during the northeast monsoon season. Furthermore, planktivorous fish displayed more ingested anthropogenic debris than the other investigated species (*p* = 0.022). Blue-coloured anthropogenic debris was commonly detected in the stomachs of fish and significantly differed between species (*p* > 0.001). Water depth and season significantly influenced the availability of food types (AF) for fish (*p* < 0.001). These findings provide evidence of the ingestion of anthropogenic debris by fish inhabiting a natural bay and signal the future anthropogenic pollution of marine fish.

## 1. Introduction

In the marine ecosystem, fish are major top predators and important for aquaculture and conservation management [1]. Fish stomach content is important primary data to directly study the feeding ecology of fish [1,2] and can be quantitatively or qualitatively presented [3,4]. To determine the feeding habits of fish, the index of relative importance (%IRI) is most frequently used by about 30% of citations examining the stomach content of fish [1]. It can be calculated from the weight or volume of a prey item and the percentages of number and frequency of occurrence [5]. It is important fundamental information to understand the functional role of the fish community in aquatic ecosystems [1,2,6] and useful to understand the interspecific interaction of resource partitioning (e.g., habitat, food) between species [7,8,9]. Fish show a narrow range of feeding adaptation, though there are some overlaps in food selection between niches in tropical estuaries [2] and nontropical estuaries [9,10,11,12,13]. There are some studies of diet overlap, food selection, and resource partitioning of fish in tropical and nontropical regions. Examples include the fish community inhabiting the bay mouth region in Thailand [14], short mackerel (*Rastrelliger brachysoma*) in a tropical estuarine environment [15], estuarine–reef habitat fish in Brazil [9], demersal fish on the continental shelf of the East/Japan Sea [10] and Southern Tyrrhenian Sea [16], deep-sea fish community in the benthic layer of the Mediterranean Sea [11], deep-sea shark (*Galeus melastomus*) in the Mediterranean Sea [12] and southern Tyrrhenian Sea [17], and diet overlap between jellyfish and juvenile fish in Alaska [13]. 

Further, fish diet composition fluctuates by season and spatiality [2], including food availability and reproductive activity [18]. Studying diets to obtain information about the food composition and feeding behaviour of species is critical to examine the ecosystem role and position of species in the ecosystem food web [19]. This information is crucial to support the management of aquatic life, especially fisheries, aquaculture, and the conservation among many species of aquatic ecosystems, in addition to supporting food security. Fish are the most important predators and have a determinant status in the trophic position of aquatic ecosystems. Many fish species play an important role in the economy of many countries around the world. Hence, information on the diet composition and feeding features of fish near Thailand is necessary. 

Fish are a useful bio-indicator of contamination of anthropogenic debris (microplastics making up the main content) to assist food security valuations [20]. However, Santana et al. [21] reported there was no evidence of plastic particle persistence in aquatic organism tissue, but consumption of fish with microplastics can lead to human health risk if toxic substances adhere to microplastics [22]. Meanwhile, consumption of microplastics can have negative effects on growth, reproduction, and survival evidence, although most of the effects are sublethal [23,24]. In addition, soft or thin plastic fragments on muddy beaches are found in higher amounts than other debris types that may have a harmful impact on marine organisms [25]. Specifically, plastic debris from anthropogenic activity occurs regardless of the season, area, or ontogenetic phase and may be passed through direct consumption and prey items [26]. 

Microplastics are defined as any plastic particle smaller than 5 mm [27] and have been widely distributed in the ocean and sediments worldwide in recent years [28], including all water of pelagic and benthic marine organisms [16,17,29,30,31,32]. Microplastics can be found in all living organisms, from tiny animals such as zooplankton [31], mysid larvae [24], and bivalves [33,34] to top predators [16,18,21,30,32,35,36,37,38,39,40,41,42]. The first report of microplastics in plankton tows was reported by Carpenter et al. [43] in North America, which later caused concern for massive water bodies [44]. For instance, microplastics have accumulated in oceans and sediment with concentrations of 3 to 102,000 m^−^^3^ and 1 to >1000 m^−^^2^, respectively [28]. On the contrary, Md Amin et al. [45] stated that the average abundance of microplastics in surface seawater of the southern South China Sea was 0.003 m^−^^3^. Microplastics pose increasing threats to the food web [38] and are transferred from prey to predator [21,31,46]. In particular, low trophic fauna is mostly affected by microplastics through ingestion [28]. For example, plastic shaped similar to algae was mistaken for food by suspension feeders [47]. When comparing the concentration of microplastics in the young and adult stages of mugilids, the early development stage of fish had a greater concentration than the older stage [37], and small-sized shellfish contained more particles than large-sized fish [34]. Therefore, there was a significant effect of prolonged exposure to microplastic harm related to age and size specific to organisms [24]. 

In the lower part of the Gulf of Thailand, some sciaenid fish show higher ingestion of mesoplastics than micro- and macroplastics [35]. Fishing net fibres were the major types of plastic found in the stomachs of some commercial marine fish, and of those, 80% were microplastics (<5 mm), whereas the rest were mesoplastics (5–25 mm) [36]. In addition, higher ingestion of microplastics was found in some commercial shrimp than in fish, most of the microplastics being fibres from textiles and fishing nets in Thailand [39]. However, the occurrence of ceramic and glass debris is greater than plastic and other debris in beach sediment due to shoreline and recreational activities [25]. 

Microplastics enter the marine environment by different pathways [42], and the ingestion of microplastics by aquatic organisms is related to their feeding habits and habitats [30]. Information on the diet composition, feeding features, and potential threats of plastic debris in marine creatures in Thailand is necessary. The present study aimed to evaluate the feeding habits of those fish and their potential contamination in environments by determining the following: (1) the occurrence of anthropogenic debris, including microplastic-like debris, in wild fish from the natural bay environment; (2) the anthropogenic debris ingestion of fish dependent upon their feeding features, water depth, and season; and (3) the variety of food types of fish at different depths or in different seasons.

## 2. Materials and Methods

### 2.1. Study Area

**Figure 1:**
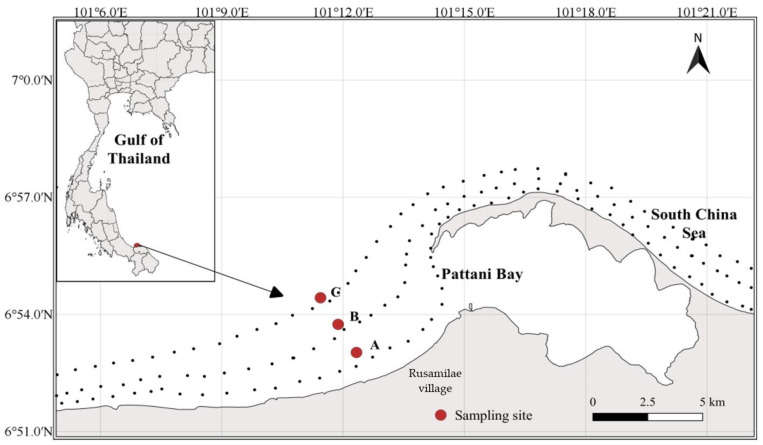
Fish sampling sites off the mouth of Pattani Bay in the lower Gulf of Thailand. The red circles represent the fish sampling sites between the water depth contours of 2, 4, and 6 m.

The survey site is situated off Pattani Bay with a surface area of 74 km^2^, located in the lower part of the Gulf of Thailand at latitude 06°52′5.3″ N and longitude 101°15′0.3″ E (Figure 1). The bay is semienclosed by a 12 km long sand spit on the northeast side. In general, the seasonal pattern of southern Thailand is influenced by the sea on both sides and heavy rains throughout the year. Based on the rainfall level, Pattani province has three seasons: dry season from January to May, moderate rainfall season (southwest monsoon) from May to September, and heavy rainy season (northeast monsoon) from September to December [14,15,48,49]. On average, Pattani province has seven months of rainfall and five months of drought due to the northeast monsoon (November–February) and southwest monsoon (May–September) [48]. The main site of this study comprised the area around the vicinity of the mouth of the bay, called Rusamilae fishing village. This area was selected because it is locally known as a major fishing ground by local fisherfolk [15,49]. 

### 2.2. Sample Collection and Storage

Altogether, 13-month fish sampling was conducted from February 2019 to February 2020. Fish were caught at three water depth contours of 2, 4, and 6 m using a set of multiple-mesh-sized mackerel gill nets with 3.0, 4.0, and 4.5 cm stretched mesh-sizes, 3.5 m deep and 540 m long; therefore, each mesh was 180 m long [14,15]. At each sampling station, a set of multiple-mesh-sized nets were hauled and left to drift for around one hour between 18:00 and 19:00. Most fish died immediately after being caught and were preserved in iceboxes as soon as possible before transportation to the laboratory at the Faculty of Science and Technology, Prince of Songkla University. Specimens were sorted and identified immediately. Fifty individual fish per species were randomly collected from each of the three sampling stations and preserved with 10% formalin for four days before being transferred to 70% ethanol for further analyses. 

### 2.3. Diet and Anthropogenic Debris Identification 

In the laboratory, a diet analysis of 5478 fish samples was performed. Total length was measured from the tip of the snout of fish to the tip of the caudal fin. Then, the fish stomach was removed from the body cavity and opened with surgical scissors. During processing, stomach content was carefully taken apart, and all identifiable prey from the 3236 nonempty stomachs were counted and specified to the lowest possible taxa with pertinent literature [50,51,52,53]. The feeding functional group was classified according to dietary preference [54]. There were seven main feeding guilds based on their %IRI: (1) planktivorous, which feeds mainly on phytoplankton and copepod zooplankton; (2) *Lucifer* feeder; (3) fish feeder, which feeds mainly on fish but also feeds on phytoplankton and copepod zooplankton; (4) *Acetes* feeder; (5) shrimp feeder; (6) piscivorous, which feeds mainly on fish; and (7) zoobenthivorous, which feeds mainly on polychaetes.

The prey types for piscivorous fish, shrimp feeder, and zoobenthivorous fish were examined under a stereomicroscope. For planktivorous fish and *Lucifer* feeder, the stomach content was put in a 15 mL measuring cylinder filled with water. The 1 mL subsample was later taken and placed on a Sedgwick Rafter chamber. Thereafter, diets were identified and counted under a light microscope. To reduce misidentification between plastic-like debris and the broken cell structure of natural prey items, nonorganic fibre was considered as plastic-like fibre, while nonorganic hard material was considered as fragments [37,42,55]. To distinguish between organic and nonorganic materials, we followed the rules of Hidalgo-Ruz [56]: Rule 1, no cellular or organic structures visible; Rule 2, fibres should be equally thick throughout their entire length; Rule 3, particles should exhibit homogeneous colour throughout the item. The hot needle test [57] was also applied for suspected cases where we were unable to distinguish between plastic and organic matter. In the presence of a hot needle, plastic pieces will melt or curl, while biological and other nonplastic materials will not. Although the aforementioned rules were applied to identify plastic materials, the whole identified fragments were not classified as totally plastic substances until they were verified by FT-IR spectrophotometer. Thus, the so-called plastic-like debris was employed for the identification of anthropogenic debris in this study.

Food types were photographed with a microscope (NIKON Eclipse E200, Nikon instruments Inc., Melville, NY, USA) attached to a digital camera (NIKON DS Fi2, Nikon instruments Inc., Melville, NY, USA). The anthropogenic debris in this study was grouped according to colour as blue, black, red, green, and white. The observed anthropogenic debris items were regarded as microplastic-like items (<5 mm), mesoplastic-like items (5–25 mm), or macroplastic-like items (>25 mm) with reference to the relevant literature [58]. 

### 2.4. Experimental Control

To avoid contamination, only laboratory glassware was used during laboratory work. To prevent sample contamination during laboratory work and visual identification, specific care was applied. To prevent contamination, an 8 cm petri dish with a few millilitres of distilled water (blank) was placed next to the working zone beside the microscope to prevent any atmospheric contamination. The results from the blank control showed no microplastic-like debris contamination.

### 2.5. Data Analysis 

Raw diet data were analysed to determine the feeding features of fish in terms of (i) the average number of food types (AF) and (ii) percentage of index of relative importance (%IRI). 

AF refers to the average number of food types observed in each stomach. Prior to estimating the %IRI of the fish, the index of relative importance (IRI) was calculated to determine the food preference of fish by the following formula [5]:

IRI_i_ = %F(%N + %V)

where %V refers to the percentage contribution of all food items in nonempty stomachs that were estimated by visual inspection and calculated based on the area covered by each prey type on a scaled Petri dish by the Hyslop formula [59]. %N and %F represent percentages of number and frequency of occurrence of prey “_i_”, respectively. Finally, %IRI was determined by the following formula [3]:

%IRI = 100 IRI_i_/∑IRI_i_

### 2.6. Statistical Analysis

Analysis of variance (ANOVA) was used to test for the ingestion of anthropogenic debris related to their feeding features, while the ingestion of debris colour in the stomach content of fish was tested. In addition, the differences of AF in fish collected from different depths and in different seasons were tested. To reduce non-normality, raw data were transformed to log (X + 1) before testing. If statistically significant, Tukey’s HSD post hoc test was then applied for the factors depth, season, feeding features, and debris colour using the R program [60].

## 3. Results

### 3.1. Food and Dominant Food Items

**Figure 2:**
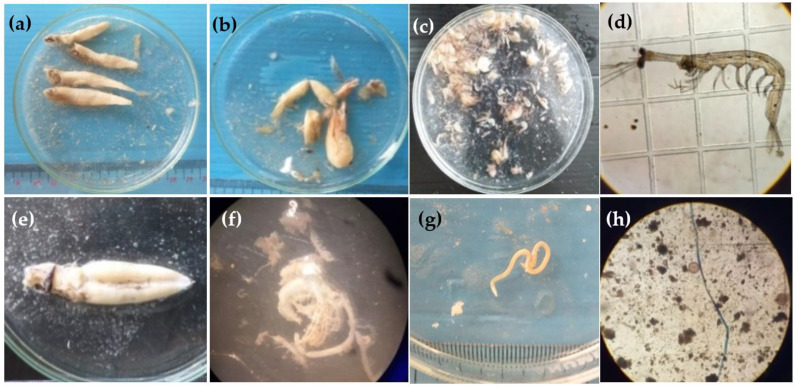
Examples of fish stomach content: (**a**) fish, (**b**) penaeid shrimp, (**c**) *Acetes* sp., (**d**) *Lucifer* sp., (**e**) squid, (**f**) hermit crab, (**g**) nematode, and (**h**) anthropogenic debris.

For data elaboration, 3236 samples of nonempty stomachs from 5478 samples of 34 fish species consisted of 22 different prey categories, which could be designated into seven main feeding guilds based on their index of relative importance (%IRI): planktivore, *Lucifer* feeder, fish feeder, *Acetes* feeder, shrimp feeder, piscivore, and zoobenthivore fishes (Table 1). Out of 34 species, only three polychaete feeders (*Nuchequula gerreoides*, *Johnius belangerii*, and *J*. *borneensis*) were recognised as zoobenthivorous fish. In this study, fish (24.1%), *Lucifer* (14.7%), and penaeid shrimp (13.4%) were the most important groups and the largest contributors for fish inhabiting the vicinity of the natural bay environment, followed by *Coscinodiscus* sp. (8.4%), copepods (8.3%), diatoms (7.6%), *Acetes* sp. (5.6%), polychaetes (5.1%), and other prey items (<3.0) (Table 2). Among planktivores, *Eubleekeria* *splendens* and *Photopectoralis* *bindus* mainly feed on diatoms with an average of 60.0% and 48.1% by %IRI, respectively. Examples of the stomach content of fish are shown in Figure 2.

The average number of food types (AF) ranged from 2 (*T. lepturus*) to 16 (*E. splendens*) types (Table 1). More than 10 food types were found mostly in planktivores, whereas fewer than 8 types were found in piscivorous fishes. The fish that had high AF were considered opportunist feeders. AF of individual fish was influenced significantly by water depth and season (*p* < 0.05), especially for most of the planktivorous fish, some *Lucifer* feeders, and one zoobenthivorous fish (*J. borneensis*) (Table 1). On the contrary, planktivorous fish such as *Anodontostoma chacunda*, *Planiliza subviridis*, some *Lucifer* feeders (*Alepes kleinii* and *A. vari*), *Acetes* feeders *(S. waitei*), shrimp feeders (*Thryssa hamiltonii*, *Panna microdon*, and *E. tetradactylum*), zoobenthivorous fish (*J. belangerii*), and piscivorous fish (*H. nehereus*, *M. cordyla*, *S. tol*, *Otolithes ruber*, *Pennahia anea*, and *T. lepturus*) showed no statistical significance, indicating their feeding was not influenced by the water depth or season. 

### 3.2. Spatial and Temporal Impacts of Depth and Season

The ingestion of anthropogenic debris by fish was influenced only by the season (*p* < 0.0001) and not by the water depth (*p* = 0.840). Tukey’s HSD post hoc test showed that high ingestion of debris was observed in the northeast monsoon season. In addition, among the anthropogenic debris ingestion of four feeding features, this was more significant for planktivorous fish (*p* = 0.022) than for the other studied fishes (Table 3). Among the five debris colours, blue was significantly more common than the others (*p* < 0.001). 

By the analysis of variance, AF of 34 fish species was influenced significantly by water depth and season (*p* < 0.001) (Table 3). Tukey’s HSD test indicated that AF significantly differed between 2 and 4 m depths. Based on the season, AF significantly differed between the dry and northeast monsoon seasons. 

### 3.3. Ingestion of Anthropogenic Debris in Fish 

**Figure 3:**
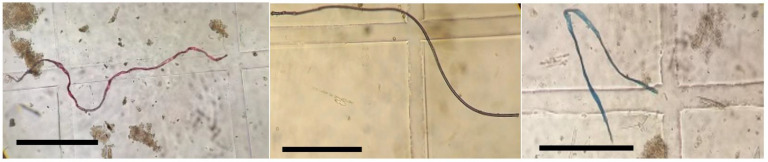
Examples of anthropogenic debris observed in the stomachs of fish. Scale bar 1 mm.

From the 5478 fish samples of 34 species, 3236 nonempty stomachs were assessed, and anthropogenic debris was observed in the guts of 12 fish species. A total of 2477 debris items were observed in the 67 guts of those 12 fish species, which accounted for 3.4% of a total of 1964 fish samples (Table 4). More debris was ingested by planktivorous fish than by piscivorous fish. Among planktivorous fish, *R*. *brachysoma* had the highest ingestion (2.6 ± 16.4 items/fish), whereas *Deveximentum insidiator* had the lowest (0.4 ± 2.5 items/fish). Compared with planktivores, *Acetes* feeder fish *Thryssa* *kammalensis* and piscivorous fish *M*. *cordyla* had low ingestion of debris at 0.02 ± 0.2 items/fish and 0.01 ± 0.1 items/fish, respectively. Examples of anthropogenic debris found in the stomachs of fish are shown in Figure 3.

Including plastic fibres and plastic bags found in the stomach content of fish, the average numbers of anthropogenic debris items are shown in Table 4. The most consumed colour of debris with a length of less than 3 mm in different species was blue, followed by green, red, black, and white, while *M*. *cordyla* had 3 cm of degraded plastic bag. Blue-coloured debris was dominant in *S*. *gibbosa*, *D*. *insidiator*, *E*. *splendens*, *P. bindus*, *P*. *subviridis*, and *R*. *brachysoma*. The green colour was dominant in *A.* *chacunda* and *Sardinella* *fimbriata*; red colour, in *H*. *kelee*; and black colour, in *Leiognathus* *equula*. 

## 4. Discussion

The present study investigated the feeding habits and anthropogenic debris ingestion of fish collected by mackerel gill nets from the natural environment off Pattani Bay, located in the southern region of the Gulf of Thailand. All fish inhabited the vicinity of the natural bay and competed for food resources, as shown by the occurrence of more than one dietary item in their stomach content. In general, most fish are opportunistic feeders and their diets may shift according to their food habitat and environmental conditions [1]. Most fish species are omnivorous in tropical estuarine environments [61], and young individual omnivorous fish of several taxa may serve as food for carnivorous fish [9]. In the nonestuarine habitat, more carnivorous or predatory fish and fewer contributions of herbivorous species were mainly observed in mangrove habitats, whilst omnivorous species dominated along the edge of mangrove and in the seagrass bed [62]. This agreed with the present study, in which most of the fish species were assigned as omnivorous, including planktivorous, *Lucifer* feeders, and *Acetes* feeders, but herbivorous fish were not observed. Pattani Bay supports a rich diverse fauna community and is an important fishing ground for local fisherfolk due to the presence of seagrass and seaweed meadows, mangrove forests, and sand–mud beds [63,64,65].

Out of 34 fish species, 19 species exhibited influences of water depth and seasonal factors on their AF by means of spatial and temporal variation of their dietary preference. In comparison within feeding features, both water depth and season were significant influence factors for most planktivorous fish, shrimp feeders, and *Lucifer* feeders, though some piscivorous fish were not affected by those factors. Particularly, fish inhabiting 2 and 4 m depths had more available prey items. It is postulated that shallow water provides more prey types for small fish, while deeper water supports larger fish (piscivorous fish).

The higher AF detected during the northeast monsoon season may be related to high rainfall and rivers (Pattani and Yamu) carrying a lot of nutrients from the land. The potential reasons for this pattern may include a recognisable seasonal trend of food availability that manifests in prey types. Consequently, this area may support the food chain of various fish feeding features. Therefore, Pattani Bay provides an important feeding ground for fish resources that should be sustained for future recruitment. The highest value of dietary items of fish might be related to the concurrence of the high abundance of prey during a specific period [18,66] during which plenty of food is derived from the land, river, and tidal mixing [61]. Some nemipterid fish showed that the AF of fish was influenced significantly by fish size classes in the lower part of the South China Sea [67].

Greater ingestion of anthropogenic debris was detected in sardines (*A*. *chacunda*, *H*. *kelee*, *S*. *fimbriata*, and *S*. *gibbosa*) than in anchovy fish (*T*. *kammalensis*). Bakun [68] stated that sardine fish are opportunist feeders whereas anchovy fish are specialists. In addition, the ingestion of anthropogenic debris was related to the filtration apparatus, as debris was ejected into the surrounding waters by the brachial system of adult fish [69]. However, Pennino et al. [70] reported that the highest microplastic ingestion was found in the lower body conditions of anchovies compared to sardines, which was in contrast to our study. In addition, de Moura and Vianna [41] reported that the ingestion of microplastics in the teleost fish was commonly fibres (20.2%) and fragments (22%). In the southern region of Thailand, some studies have been conducted on plastic debris in commercial fish and shrimp species [25,35,36,39,71]. Compared with the present study, there were different results depending on the study focus by species. For instance, there was no presence of anthropogenic debris in some sciaenid fish in this study, in contrast to the report by Azad et al. [35]. Moreover, the ingestion of plastic debris in *S*. *gibbosa* (0.3 items/fish), *E*. *splendens* (1 item/fish), and *R*. *brachysoma* (1 item/fish) from Azad et al. [36] was lower than that in the present study, while the ingestion of plastic debris in *M*. *cordyla* (1.6 items/fish) was lower than that in the aforementioned publication. The differences likely depend on feeding habitat, fishing ground, and seasonally available food. Season is a significant factor in this habitat, with especially high ingestion of plastic debris during the northeast monsoon season, but not irrespective of water depth. This may be related to the seasonal river inflow that carries plastic contaminants during the rainy season [20,72]. In addition, Barletta et al. [20] reported that the concentration of microplastic debris was higher in upstream locations during the dry season, while seaward areas had higher concentrations during the rainy season.

Hajisamae et al. [2] concluded that carnivorous fish depend mostly on their visual ability for prey detection. This was in agreement with the present study; the occurrence of anthropogenic debris ingestion was high among planktivorous fish, and *R*. *brachysoma* had especially high amounts of ingested anthropogenic debris. Therefore, it may be assumed that the ingestion of plastic debris may depend on the feeding behaviour of an individual species. In addition, Lima et al. [72] reported that planktivorous fish might ingest microplastics along with their food and then transfer them to larger predators. Klangnurak and Chunniyom [73] reported that microplastic accumulation in the gastrointestinal tracts of pelagic and demersal fishes showed no significant differences indicating the potential threats of microplastics throughout the water columns. On the contrary, Jabeen et al. [74] stated that the ingestion of plastic items in fish was closely related to the habitat and the gastrointestinal tract structure (such as intestine and stomach). However, Borges-Ramírez [22] reported that high ingestion of microplastics was detected in demersal fish species compared to pelagic fish. Meanwhile, omnivorous fish showed higher ingestion of MPs compared to herbivorous and carnivorous fish [32]. Therefore, future work on microplastic ingestion by fish should include the entire gastrointestinal tract and digestion process and then be extended to compare surface water with substrata. Among microplastic types including fragment, foam, fibre, film pellet, and others, the first was dominant and accounted for 42% with an average size of 3.72 ± 4.70 mm in the Yellow Sea [75]. In Taiwan, 91% of the microplastics found in common seafood species (shrimp, crab, oyster, and clam) were plastic fragments [31]. For comparison of plastic debris size, Núñez et al. [76] examined the distribution of microplastics across Galápagos Island. It was found that the size range of 0.15–0.5 mm was dominant in 100% of the water samples and marine organisms [76], and this is smaller than our result of mostly 1–3 mm in length with the exception of degraded plastic bag in *M*. *cordyla* that was 3 cm size in the stomach content of fish.

Most of the anthropogenic debris found in the present study was blue in colour, and the contributions differed significantly by debris type. According to a report by Pradit et al. [25], there was more blue-coloured debris in the mudflats than at beach sites. This finding corresponds with the bottom characteristics of Pattani Bay (sandy–muddy), which is a semienclosed bay located in the lower Gulf of Thailand, facing the South China Sea. Blue-coloured anthropogenic debris was ingested at the highest rate by *R*. *brachysoma*. This could be related to the utilisation of fishing gear and fishery activities where this species is intentionally caught by local fisherfolk in this fishing ground. Moreover, de Sa et al. [77] reported that the presence of microplastics in natural waters moves with water movement; therefore, it seems similar to natural prey, which leads to fish facing food selection difficulties. In addition, the size of plastic particles varied according to their colour, including white, tan, and yellow plastics; in particular, white colour plastic, reduced in size, was similar to prey for some planktivorous fishes [78]. Teleosts and elasmobranch fish mostly ingested blue microplastics [41], which was similar to the findings of the present study of teleost fish.

## 5. Conclusions

AF varied according to water depth and season; in particular, there were more available prey types at 2 and 4 m depths for fish. Along with food consumption by fish, anthropogenic debris ingestion differed by feeding features, though it was especially high in planktivorous fish. The ingestion of plastic debris by colour also differed by fish species, with especially high ingestion of blue-coloured plastics. Our study provides evidence of plastic pollutant ingestion by fish inhabiting the vicinity of Pattani Bay and alerts for the potential effect of these pollutants on the trophic web. Further studies are urgently needed to verify plastic debris using FT-IR spectrophotometry and investigate the contamination of fish from different water columns and substrates, and the investigation of the stomach content of fish should be extended to pursue a better understanding of the effects of plastic debris contamination on the marine trophic web.

## Figures and Tables

**Table 1:** Feeding habit and occurrence of anthropogenic debris by fish species off Pattani Bay, in the lower Gulf of Thailand (AF = number of food types; bold indicating statistically significant *p* value). Parentheses enclose the average number of fish sampled (N) per month. * Statistical analysis applied with month factor when seasonal factor was not available; ** anthropogenic debris present.

Family	Species	Sample (N)	Nonempty Stomach	Total Length (Mean ± SD)	AF (Mean ± SD)	Depth	Season	Feeding Features
						*p* Value		
Clupeidae	*Anodontostoma chacunda*	625 (48.1)	193	12.9 ± 1.3	13 (1.7 ± 1.3)	0.779	0.611	Planktivore **
	*Hilsa kelee*	136 (22.7)	110	15.3 ± 1.7	13 (3.3 ± 1.3)	**0.0001**	**<0.0001**	Planktivore **
	*Sardinella fimbriata*	211 (30.1)	181	13.7 ± 0.8	14 (2.3 ± 1.2)	**0.036**	0.071	Planktivore **
	*S. gibbosa*	231 (30.4)	218	13.5 ± 1.6	13 (1.7 ± 1.5)	**0.007**	**<0.0001**	*Lucifer* feeder **
Engraulidae	*Setipinna taty*	185 (20.6)	49	13.9 ± 1.5	10 (1 ± 0.8)	**0.0008**	**0.032**	*Lucifer* feeder
	Stolephorus commersonnii *	18 (4.5)	8	9.6 ± 1.9	5 (0.9 ± 0.4)	**<0.0001**	0.588	Fish feeder
	*S. waitei*	19 (4.8)	6	8.5 ± 2.0	7 (1.5 ± 1.9)	0.586	0.358	*Acetes* feeder
	*Thryssa hamiltonii*	325 (25)	168	17.9 ± 1.8	9 (1.0 ± 0.4)	0.791	0.075	Shrimp feeder
	*T. kammalensis*	148 (21.1)	43	9.9 ± 0.9	10 (0.9 ± 0.7)	**0.036**	0.059	*Acetes* feeder **
	*T. setirostris*	59 (14.7)	10	14.6 ± 0.9	4 (0.9 ± 0.3)	**<0.0001**	**<0.0001**	Shrimp feeder
Chirocentridae	*Chirocentrus nudus*	29 (5.8)	27	29.9 ± 4.2	6 (1.0 ± 0.3)	**0.019**	**0.0001**	Piscivore
Pristigasteridae	*Opisthopterus tardoore*	293 (22.5)	100	14.8 ± 1.6	8 (0.8 ± 0.4)	**0.002**	**0.046**	*Lucifer* feeder
Synodontidae	Harpadon nehereus *	116 (38.7)	22	21.1 ± 1.6	3 (0.9 ± 0.4)	0.755	0.763	Piscivore
Carangidae	*Alepes kleinii*	243 (20.3)	121	12.0 ± 1.9	8 (0.7 ± 0.5)	0.295	0.239	*Lucifer* feeder
	*A. vari*	14 (2.3)	10	13.1 ± 1.6	3 (0.8 ± 0.4)	0.072	1.000	*Lucifer* feeder
	*Megalaspis cordyla*	184 (15.3)	159	15.6 ± 2.3	7 (0.9 ± 0.5)	0.559	0.113	Piscivore **
	*Scomberoides tol*	23 (3.8)	18	15.4 ± 2.4	5 (0.9 ± 0.4)	0.664	0.891	Piscivore
Leiognathidae	*Deveximentum insidiator*	102 (7.8)	71	9.8 ± 2.5	10 (1.0 ± 1.2)	0.312	**<0.001**	*Lucifer* feeder **
	Eubleekeria jonesi *	40 (20)	34	7.3 ± 0.8	9 (1.0 ± 1.7)	**<0.001**	**<0.001**	Planktivore
	*E. splendens*	272 (20.9)	146	7.5 ± 1.0	16 (1.7 ± 1.4)	**0.003**	**0.023**	Planktivore **
	*Leiognathus equula*	76 (5.8)	61	9.2 ± 0.8	11 (0.6 ± 1.0)	**0.002**	0.098	Planktivore **
	*Nuchequula gerreoides*	41 (20.5)	31	8.9 ± 1.2	6 (0.7 ± 0.9)	**0.002**	0.098	Zoobenthivore
	*Photopectoralis bindus*	319 (26.6)	191	9.4 ± 1.1	12 (0.5 ± 0.9)	0.100	**0.009**	Planktivore **
Sciaenidae	*Dendrophysa russelii*	65 (6.5)	32	12.3 ± 1.6	8 (0.7 ± 0.6)	**0.048**	0.371	Shrimp feeder
	*Johnius belangerii*	68 (13.6)	35	14.7 ± 1.1	6 (0.7 ± 0.6)	0.335	0.076	Zoobenthivore
	*J. borneensis*	167 (18.6)	89	15.1 ± 1.3	9 (0.7 ± 0.6)	**0.011**	**0.001**	Zoobenthivore
	*Otolithes ruber*	131 (13.1)	36	18.3 ± 2.1	6 (0.9 ± 0.5)	0.064	0.284	Piscivore
	*Panna microdon*	62 (8.9)	39	20.6 ± 3.3	5 (0.8 ± 0.5)	0.926	0.299	Shrimp feeder
	*Pennahia anea*	76 (15.2)	12	14.0 ± 1.6	3 (0.9 ± 0.5)	0.611	0.426	Piscivore
Polynemidae	*Eleutheronema tetradactylum*	72 (6)	53	21.9 ± 2.6	5 (1.1 ± 0.4)	0.997	0.433	Shrimp feeder
Mugilidae	*Planiliza subviridis*	132 (14.7)	39	17.7 ± 2.0	10 (0.8 ± 1.2)	0.542	0.096	Planktivore **
Trichiuridae	*Trichiurus lepturus*	62 (8.9)	28	44.1 ± 5.3	2 (1.0 ± 0.0)	0.427	0.431	Piscivore
Scombridae	*Rastrelliger brachysoma*	554 (42.6)	552	16.4 ± 1.9	15 (3.8 ± 1.2)	**0.031**	**0.003**	Planktivore **
	*Scomberomorus commerson*	380 (29.2)	344	21.1 ± 4.3	7 (1.0 ± 0.2)	**0.033**	0.799	Piscivore

**Table 2:** Index of relative importance (%IRI) contribution of anthropogenic debris and other dietary types of fishes inhabiting the natural bay environment (Deb = anthropogenic debris; Fish = fish; Luc = *Lucifer* sp.; Shri = shrimp; Ace = *Acetes* sp.; Cop = copepods; other = other zooplankton; Poly = polychaetes; Cosc = *Coscinodiscus* sp.; Diat = other diatoms, Dino = dinoflagellates; Stom = stomatopods; Squid = squid; Cru = other crustaceans excluding copepods; Crab = crabs; Sagi = *Sagitta* sp.; Brit = brittle stars; Sea = sea urchins; Moll = molluscs; Amp = amphipods; Nem = nematodes; Mis = miscellaneous). In bold: main food contribution.

Species	Deb	Fish	Luc	Shri	Acet	Cop	other	Poly	Nem	Cosc	Diat	Dino	Stom	Squid	Crus	Crab	Sagi	Bri	Sea	Moll	Amp	Mis
*A. chacunda*	1.7	0.0	0.0	0.0	0.0	8.3	**38.0**	1.2	7.5	25.5	11.9	3.4	0.0	0.0	0.4	0.0	0.0	0.0	0.0	1.4	0.0	0.6
*H. kelee*	0.1	0.0	0.0	1.0	0.0	**22.7**	2.1	0.3	0.1	37.0	23.3	12.6	0.0	0.0	0.3	0.0	0.0	0.0	0.0	0.4	0.0	0.1
*S. fimbriata*	2.5	0.0	11.6	2.4	0.0	**45.5**	0.1	1.4	0.7	16.8	15.8	1.1	0.0	0.0	0.0	0.5	0.0	0.0	0.0	1.4	0.0	0.1
*S* *. gibbosa*	0.0	0.0	**35.5**	0.3	0.0	16.8	1.1	3.4	0.0	23.3	15.2	3.4	0.0	0.0	0.1	0.2	0.0	0.0	0.0	0.6	0.0	0.1
*S. taty*	0.0	0.0	**61.4**	21.9	7.1	0.0	0.0	3.6	0.0	1.7	0.0	0.0	0.0	0.0	3.4	0.0	0.0	0.0	0.0	0.9	0.0	0.0
*S. commersonnii*	0.0	**57.1**	14.3	0.0	0.0	14.3	0.0	0.0	0.0	14.3	0.0	0.0	0.0	0.0	0.0	0.0	0.0	0.0	0.0	0.0	0.0	0.0
*S* *. waitei*	0.0	0.0	17.5	0.0	**36.8**	20.2	0.0	0.0	0.0	18.4	7.0	0.0	0.0	0.0	0.0	0.0	0.0	0.0	0.0	0.0	0.0	0.0
*T. hamiltonii*	0.0	33.2	2.0	**33.5**	18.1	0.0	8.6	0.7	0.0	0.0	0.0	0.0	2.8	0.0	0.0	0.0	0.0	0.0	0.0	1.2	0.0	0.0
*T* *. kammalensis*	3.8	0.0	0.0	30.8	**50.0**	0.0	0.0	0.0	0.0	3.8	0.0	0.0	0.0	0.0	0.0	0.0	0.0	0.0	0.0	0.0	11.5	0.0
*T* *. setirostris*	0.0	0.0	22.2	**44.4**	0.0	0.0	0.0	0.0	0.0	0.0	0.0	0.0	33.3	0.0	0.0	0.0	0.0	0.0	0.0	0.0	0.0	0.0
*C. nudus*	0.0	**79.4**	0.0	15	1.8	0.0	0.0	0.0	0.0	0.0	0.0	0.0	0.0	3.8	0.0	0.0	0.0	0.0	0.0	0.0	0.0	0.0
*O. tardoore*	0.0	2.8	**47.2**	25.5	22.4	0.2	0.0	0.0	0.0	0.1	0.0	0.0	0.0	0.0	0.0	0.0	0.0	0.0	1.7	0.0	0.0	0.0
*H. nehereus*	0.0	**89.5**	0.0	10.5	0.0	0.0	0.0	0.0	0.0	0.0	0.0	0.0	0.0	0.0	0.0	0.0	0.0	0.0	0.0	0.0	0.0	0.0
*A. kleinii*	0.0	0.0	**87.0**	0.8	6.8	0.0	0.0	2.3	0.0	0.0	0.0	0.0	0.0	0.0	1.7	0.0	0.0	0.0	1.4	0.0	0.0	0.0
*A* *. vari*	0.0	0.0	**100.0**	0.0	0.0	0.0	0.0	0.0	0.0	0.0	0.0	0.0	0.0	0.0	0.0	0.0	0.0	0.0	0.0	0.0	0.0	0.0
*M. cordyla*	1.2	**80.9**	1.5	0.8	10.3	0.0	4.6	0.0	0.0	0.0	0.0	0.0	0.0	0.0	0.4	0.0	0.0	0.0	0.0	0.4	0.0	0.0
*S. tol*	0.0	**57.7**	20.0	2.3	20.0	0.0	0.0	0.0	0.0	0.0	0.0	0.0	0.0	0.0	0.0	0.0	0.0	0.0	0.0	0.0	0.0	0.0
*D. insidiator*	3.0	0.0	**53.0**	0.0	2.8	12.6	0.0	2.8	0.0	7.4	13.7	1.4	0.0	0.0	0.0	3.5	0.0	0.0	0.0	0.0	0.0	0.0
*E. jonesi*	0.0	0.0	3.5	0.0	0.0	**36.1**	4.6	0.0	2.6	36.9	4.7	0.0	0.0	0.0	1.3	0.0	0.0	0.0	0.0	10.3	0.0	0.0
*E* *. splendens*	2.6	0.0	1.6	0.0	0.0	11.2	7.2	1.3	0.7	8.3	**60.0**	2.7	0.0	0.0	0.1	0.2	1.0	0.0	0.8	0.4	0.0	1.8
*L. equula*	7.9	0.0	11.4	0.0	0.0	**22.5**	1.8	16.4	0.3	4.9	24.1	4.3	0.0	0.0	1.3	0.0	0.0	0.0	0.0	0.0	0.0	5.2
*N. gerreoides*	0.0	0.0	0.0	0.0	0.0	2.6	2.8	**33.8**	0.0	28.4	0.0	0.0	0.0	0.0	0.8	0.0	0.0	0.0	0.0	9.9	21.5	0.0
*P. bindus*	10.1	0.0	8.5	0.0	0.0	10.8	0.9	7.4	0.3	1.9	**48.1**	0.6	0.0	0.0	0.3	0.0	1.7	0.6	0.0	0.4	0.8	7.7
*D. russelii*	0.0	20.3	0.0	**50.3**	5.9	0.0	0.0	5.9	0.6	0.0	0.0	0.0	0.0	5.9	0.0	8.2	0.0	0.0	0.0	0.0	0.0	2.9
*J. belangerii*	0.0	7.1	0.0	30.2	0.0	0.0	0.0	**37.7**	0.0	0.0	0.0	0.0	0.0	0.0	17.9	7.1	0.0	0.0	0.0	0.0	0.0	0.0
*J* *. borneensis*	0.0	15.7	0.0	15.0	0.0	0.0	0.0	**50.8**	1.4	0.0	0.0	0.0	0.0	0.0	3.5	7.9	0.0	0.5	0.0	3.9	0.0	1.4
*O. ruber*	0.0	**50.0**	0.0	32.1	5.3	0.0	0.0	0.0	0.0	0.0	0.0	0.0	3.6	3.6	0.0	0.0	0.0	0.0	0.0	0.0	0.0	5.4
*P. microdon*	0.0	13.5	0.0	**63.6**	0.0	0.0	0.0	2.1	0.0	0.0	0.0	0.0	0.0	0.0	0.0	2.1	0.0	0.0	0.0	0.0	0.0	18.6
*P. anea*	0.0	**87.3**	0.0	12.7	0.0	0.0	0.0	0.0	0.0	0.0	0.0	0.0	0.0	0.0	0.0	0.0	0.0	0.0	0.0	0.0	0.0	0.0
*E. tetradactylum*	0.0	25.3	0.0	**71.6**	1.4	0.0	0.0	0.0	0.0	0.0	0.0	0.0	1.7	0.0	0.0	0.0	0.0	0.0	0.0	0.0	0.0	0.0
*P. subviridis*	5.0	0.0	0.0	0.0	0.0	10.6	13.8	1.8	1.2	**35.5**	16.2	4.9	0.0	0.0	0.5	0.0	0.0	0.0	0.0	1.5	0.0	8.9
*T. lepturus*	0.0	**100.0**	0.0	0.0	0.0	0.0	0.0	0.0	0.0	0.0	0.0	0.0	0.0	0.0	0.0	0.0	0.0	0.0	0.0	0.0	0.0	0.0
*R. brachysoma*	0.4	0.0	1.3	0.1	0.0	**47.3**	0.9	0.3	0.1	20.9	17.0	8.0	0.0	0.0	0.3	0.2	0.0	0.0	0.0	2.9	0.0	0.3
*S. commerson*	0.0	**99.4**	0.0	0.5	0.0	0.0	0.0	0.0	0.0	0.0	0.0	0.0	0.0	0.0	0.0	0.0	0.0	0.0	0.0	0.0	0.0	0.0
Average	1.1	24.1	14.7	13.4	5.6	8.3	2.5	5.1	0.5	8.4	7.6	1.2	1.2	0.4	1.0	0.9	0.1	0.03	0.1	1.0	1.0	1.6

**Table 3:** Results of analysis of variance on the number of food types and ingestion of anthropogenic debris in 34 fish species by depth and season in addition to feeding features (df = degrees of freedom, MS = mean sum of squares).

	Factor	df	MS	F Value	*p* Value
Ingestion of anthropogenic debris in fish	Depth (d)	2	0.02	0.17	0.840
	Season (s)	2	1.25	12.97	<0.0001
	d × s	4	0.17	1.76	0.135
Ingestion of anthropogenic debris in four feeding features	Feeder (f)	3	1.21	3.23	<0.022
Colour of anthropogenic debris in fish stomach	Debris colour (c)	4	4.05	5.38	<0.001
Food items (AF)	Depth (d)	2	1.13	3.98	0.019
	Season (m)	2	10.99	38.67	<0.0001
	d × s	4	1.49	5.23	<0.001

**Table 4:** Abundance of ingested anthropogenic debris by number and colour in the 12 marine fish species of non-empty-stomach fish. Parentheses enclose the number of fish with ingested debris.

Species	Examined Fish	% of Debris in Fish	No. of Items	Items/Fish	Length of Debris (mm)	Blue	Green	Red	Black	White
*A* *. chacunda*	193 (9)	4.7	195	1.00 ± 4.9	1–3	0	170	25	0	0
*H* *. kelee*	110 (3)	2.7	60	0.50 ± 3.5	<1.0	10	20	30	0	0
*S* *. fimbriata*	181 (4)	2.2	120	0.70 ± 5.9	1–3	0	120	0	0	0
*S* *. gibbosa*	218 (4)	1.8	135	0.60 ± 5.6	1–3	75	0	60	0	0
*T* *. kammalensis*	43 (1)	2.3	1	0.02 ± 0.2	1–2	1	0	0	0	0
*M* *. cordyla*	159 (1)	0.6	1	0.01 ± 0.1	3 cm	0	0	0	0	1
*D* *. insidiator*	71 (2)	2.8	30	0.40 ± 2.5	1–2	20	10	0	0	0
*E* *. splendens*	146 (12)	8.2	210	1.40 ± 5.1	<1.0–2.0	80	35	25	70	0
*L* *. equula*	61 (3)	4.9	45	0.70 ± 3.3	1–2	10	0	15	20	0
*P* *. bindus*	191 (9)	4.7	180	0.90 ± 4.5	<1.0–2.0	120	0	60	0	0
*P* *. subviridis*	39 (2)	5.1	45	1.20 ± 5.3	<1.0	45	0	0	0	0
*R* *. brachysoma*	552 (17)	3.1	1455	2.60 ± 16.4	0.5–3	955	260	240	0	0
Total	1964 (67)	3.4	2477	1.30 ± 9.5	-	1316	615	455	90	1

## Data Availability

The raw data supporting the statistical analysis in this article will be made available by the first author.
